# RETIREMENT FINANCIAL LITERACY LEVELS AMONG JAMAICANS

**DOI:** 10.1093/geroni/igz038.2151

**Published:** 2019-11-08

**Authors:** Kayon Donaldson-Davis

**Affiliations:** Mona Ageing & Wellness Centre, Kingston, Jamaica

## Abstract

Although the general literacy rate (ability to read and write at a 6th-grade level or higher) of Jamaicans is 89%, the number of people who are adequately prepared financially is not comparable. Dissertation research findings revealed that 52% (n=203) of respondents had not received any financial education. Approximately 71% of respondents who reported high levels of financial distress and low financial well-being, had not received financial information about retirement planning. Results from the 2012 Social Status on the Elderly in Jamaica showed that 60.5% (n=1,716) of respondents had no pension and of those receiving money from abroad, most (75.0%, n=813) indicated that they received it only occasionally. Those who were self-employed, women and rural residents were most likely not to have a pension. This paper describes financial literacy levels among Jamaicans using two separate data sets – the 2012 Social Status on the Elderly and the 2018 Financial Preparation Study.

